# Supplementary material for: Quantitative imaging of RAD51 expression as a marker of platinum resistance in ovarian cancer
Source: EMBO Mol Med. 2021 Mar 11;13(5):e13366. doi: 10.15252/emmm.202013366 (PMC8103098; doi:10.15252/emmm.202013366)
Supplement: Supplementary file 1 — Appendix [file EMMM-13-e13366-s001.pdf]

Appendix

Table of Contents

Appendix Table S1. Multivariate analysis of continuous $RAD51_{NES}$ and Ki67 extent as a predictor of PFS and OS in HRD negative cases of the SCOTROC4 cohort (Cox proportional hazards model). 2

Appendix Table S2. Clinicopathologic characteristics of HGSOC patients of the BCC cohort used in this study. 3

Appendix Table S3. Clinicopathologic characteristics of patients of the SCOTROC4 cohort used in this study. 4

Appendix Table S4. Antibodies used in fluorescent immunohistochemistry experiments in this study.... 5

Appendix Table S1. Multivariate analysis of continuous $RAD51_{NES}$ and Ki67 extent as a predictor of PFS and OS in HRD negative cases of the SCOTROC4 cohort (Cox proportional hazards model).

Variable		Total cases (*n*=122) missing values (*n*=51)			
		PFS		OS	
		HR (95% CI)	p-value	HR (95% CI)	*p*-value
$RAD51_{NES}$ (continuous)		1.2 (0.95 to 1.6)	0.122	1.6 (1.1 to 2.2)	0.012
Ki67% (continuous)		1.0 (0.98 to 1.0)	0.777	0.99 (0.96 to 1.0)	0.462
Age					
	<65	Ref.		Ref.	
	≥65	1.1 (0.63 to 1.9)	0.714	1.4 (0.65 to 2.9)	0.401
Stage			<0.001		0.029
	I	Ref.		Ref.	
	II	3.2 (0.74 to 14.2)	0.120	11.0 (1.1 to 108.9)	0.040
	III	11.2 (3.1 to 40.6)	<0.001	12.5 (1.4 to 108.3)	0.022
	IV	5.9 (1.4 to 24.0)	0.014	5.0 (0.51 to 48.8)	0.168
Histology			0.293		0.001
	Serous	Ref.		Ref.	
	Mucinous	3.7 (0.97 to 14.1)	0.056	19.2 (4.0 to 91.9)	<0.001
	Clear cell	2.6 (0.66 to 10.1)	0.175	17.8 (3.6 to 87.7)	<0.001
	Endometroid	1.5 (0.64 to 3.3)	0.370	1.2 (0.40 to 3.8)	0.731
	Other	1.0 (0.13 to 7.6)	0.983	no cases	
Grade (differentiation)					
	1 - well	Ref.		Ref.	
	2 - moderate and 3 - poor	2.6 (0.91 to 7.6)	0.074	7.8 (0.97 to 62.9)	0.053
Performance status			0.492		0.246
	0	Ref.		Ref.	
	1	1.2 (0.64 to 2.3)	0.542	1.3 (0.53 to 3.2)	0.553
	2 and 3	1.6 (0.72 to 3.7)	0.237	2.5 (0.78 to 7.8)	0.124
Bulk of residual disease			0.056		0.279
	None/microscopic	Ref.		Ref.	
	Macroscopic < 2 cm	2.2 (1.2 to 4.1)	0.017	2.1 (0.84 to 5.0)	0.113
	Macroscopic > 2 cm	2.0 (0.96 to 4.2)	0.066	1.6 (0.58 to 4.4)	0.372

$RAD51_{NES}$	RAD51 nuclear expression score
PFS	progression-free survival
OS	overall survival
HR	Hazard ratio
CI	Confidence interval
HRD	Homologous Recombination Deficiency score
Ref.	Reference sample

Appendix Table S2. Clinicopathologic characteristics of HGSOC patients of the BCC cohort used in this study.

		All cases	RAD51-High (Q4)	RAD51-IQR (Q2+3)	RAD51-Low (Q1)
		n (%)	*n* (%)	*n* (%)	*n* (%)
Total		284 (100%)	71 (25%)	142 (50%)	71 (25%)
Age					
	< 65 years	166 (58.5%)	43 (15.1%)	75 (26.4%)	48 (16.9%)
	≥ 65 years	118 (41.5%)	28 (9.8%)	67 (23.6%)	23 (8.1%)
Stage					
	I	10 (3.5%)	1 (0.3%)	3 (1.1%)	6 (2.1%)
	II	17 (6.0%)	5 (1.8%)	9 (3.2%)	3 (1.1%)
	III	229 (80.6%)	56 (19.7%)	118 (41.5%)	55 (19.4%)
	IV	26 (9.2%)	9 (3.2%)	11 (3.9%)	6 (2.1%)
	no data	2 (0.7%)	0 (0.0%)	1 (0.3%)	1 (0.3%)

Appendix Table S3. Clinicopathologic characteristics of patients of the SCOTROC4 cohort used in this study.

	All cases *n* (%)	RAD51-High (Q4) *n* (%)	RAD51-IQR (Q2+3) *n* (%)	RAD51-Low (Q1) *n* (%)
Total	268 (100%)	67 (25.0%)	134 (50.0%)	67 (25.0%)
Age				
< 65 years	126 (47.0%)	41 (15.3%)	57 (21.3%)	43 (16.0%)
≥ 65 years	142 (53.0%)	26 (9.7%)	77 (28.7%)	24 (9.0%)
Stage				
I	39 (14.6%)	6 (2.2%)	14 (5.2%)	19 (7.1%)
II	26 (9.7%)	7 (2.6%)	10 (3.7%)	9 (3.4%)
III	168 (62.7%)	42 (15.7%)	92 (34.3%)	34 (12.7%)
IV	35 (13.1%)	12 (4.5%)	18 (6.7%)	4 (1.5%)
Histology				
Serous	210 (78.4%)	63 (23.5%)	109 (40.7%)	38 (14.2%)
Mucinous	13 (4.9%)	1 (0.4%)	2 (0.7%)	10 (3.7%)
Clear cell	12 (4.5%)	1 (0.4%)	2 (0.7%)	9 (3.4%)
Endometroid	27 (10.1%)	2 (0.7%)	15 (5.6%)	10 (3.7%)
Other	6 (2.2%)	0 (0.0%)	6 (2.2%)	0 (0.0%)
Grade (differentiation)				
1 – well	20 (7.5%)	2 (0.7%)	9 (3.4%)	9 (3.4%)
2 – moderate	51 (19.0%)	12 (4.5%)	25 (9.3%)	14 (5.2%)
3 – poor	142 (53.0%)	40 (14.9%)	69 (25.7%)	33 (12.3%)
no data	55 (20.5%)	13 (4.9%)	31 (11.6%)	11 (4.1%)
Performance status				
0	89 (33.2%)	18 (6.7%)	47 (17.5%)	24 (9.0%)
1	137 (51.1%)	41 (15.3%)	63 (23.5%)	33 (12.3%)
2	41 (15.3%)	8 (3.0%)	24 (9.0%)	9 (3.4%)
3	1 (0.4%)	0 (0.0%)	0 (0.0%)	1 (0.4%)
Bulk of residual disease				
None/microscopic	106 (39.6%)	22 (8.2%)	48 (17.9%)	36 (13.4%)
Macroscopic < 2cm	89 (33.2%)	27 (10.1%)	43 (16.0%)	19 (7.1%)
Macroscopic > 2cm	55 (20.5%)	13 (4.9%)	34 (12.7%)	8 (3.0%)
no data	18 (6.7%)	5 (1.9%)	9 (3.4%)	4 (1.5%)
HRD score				
HRD positive	67 (25.0%)	18 (6.7%)	35 (13.1%)	14 (5.2%)
HRD negative	173 (64.6%)	41 (15.3%)	83 (31.0%)	19 (7.1%)
no data	28 (10.4%)	8 (3.0%)	16 (6.0%)	4 (1.5%)

Appendix Table S4. Antibodies used in fluorescent immunohistochemistry experiments in this study.

Antibody	clone	source	clonality	Antigen retrieval buffer pH	dilution used for fIHC
anti-RAD51	EPR4030(3)	Epitomix/Abcam	rabbit monoclonal	9	1:4000 (tumour)<br>1:8000 (cell block)
anti-EpCAM	C-10	Santa Cruz	mouse monoclonal	6	1:250
anti-Ki67	MIB-1	Dako	mouse monoclonal	6	1:100
anti-phospho-Histone H2A.X (Ser139) (γH2AX)	JBW301	Upstate/Millipore	mouse monoclonal	9	1:1000
anti-CD3	AE1/AE3	Dako	mouse monoclonal	9	1:200
anti-CD8	4B11	Leica	mouse monoclonal	9	1:100
anti-FOXP3	236A/E7	Abcam	mouse monoclonal	9	1:200
anti-CD163	MRQ-26	Cell Marque	mouse monoclonal	9	1:200
anti-cytokeratin	-	Dako (DKO.A045201)	rabbit polyclonal	9	1:200
