# Supplementary material for: Quantitative imaging of RAD51 expression as a marker of platinum resistance in ovarian cancer
Source: EMBO Mol Med. 2021 Mar 11;13(5):e13366. doi: 10.15252/emmm.202013366 (PMC8103098; doi:10.15252/emmm.202013366)
Supplement: Supplementary file 2 — Expanded View Figures PDF [file EMMM-13-e13366-s007.pdf]

Expanded View Figures

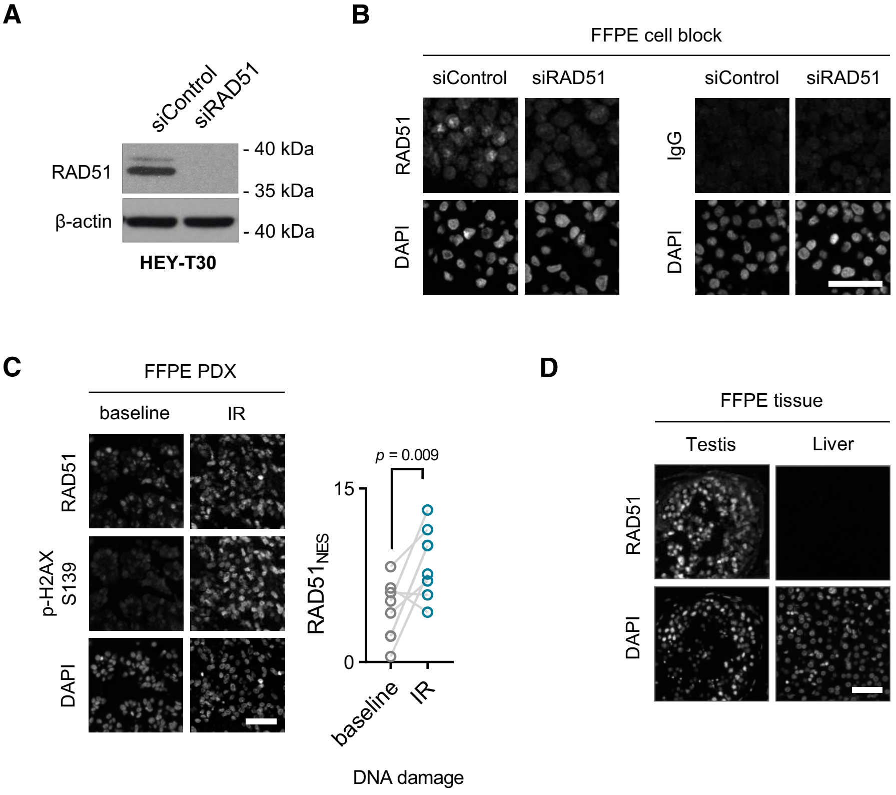

Figure EV1. Validation of a RAD51-specific antibody.

- A Immunoblot of RAD51 in HEY-T30 control and knock-down cell lines. The EPR4030(3) antibody reveals a single band of 37 kDa size in control cells (top band results from post-translational modification of RAD51), which is not present in RAD51 knock-down cells. Blot is a representation of three independent experiments.
- B Cells from (A) were used to create a FFPE cell block used for RAD51 fluorescent IHC (*left*). Cells from (A) in an FFPE block stained with an IgG isotype control (*right*). Scale bar is 50μm. All images are representative of three independent experiments.
- C Fluorescent IHC FFPE block of an ovarian PDX treated *ex vivo* with γ-radiation and stained for RAD51 and p-H2AX S139 (left). Scale bar is 50 μm. $RAD51_{NES}$ score for seven ovarian PDXs before and after treatment with γ-radiation (IR) (right). Paired *t*-test.
- D RAD51 fluorescent IHC on normal FFPE tissues. Testis is shown as a positive control and liver a negative control. Scale bar is 50 μm. All images are representative of three independent biological samples.

Figure EV2. Correlation of Ki67 % extent and HRD phenotype with $RAD51_{NES}$.

- A Correlation of Ki67 % extent and $RAD51_{NES}$ in the BCC cohort. Spearman correlation (*left*) and one-way ANOVA with Bonferroni correction (*right*). Median with interquartile range.
- B Kaplan–Meier plots for PFS (*left*) and OS (*right*) stratified according to Ki67 extent quartile in the BCC cohort. Q—quartile. Log-rank test, shading denotes 95% confidence intervals.
- C Correlation of Ki67 extent and $RAD51_{NES}$ in the SCOTROC4 cohort. Spearman correlation (*left*) and one-way ANOVA with Bonferroni correction (*right*). Median with interquartile range.
- D Kaplan–Meier plots for PFS (*left*) and OS (*right*) stratified according to Ki67 extent quartile in the SCOTROC4 cohort. Q—quartile. Log-rank test, shading denotes 95% confidence intervals.
- E Correlation of $RAD51_{NES}$ with *BRCA* mutation status in EOC. One-way ANOVA. Median with interquartile range.
- F Linear regression of "genomic scar" HRD score assay and $RAD51_{NES}$. Vertical dashed line denotes HRD positivity score of 42.

© 2021 The Authors

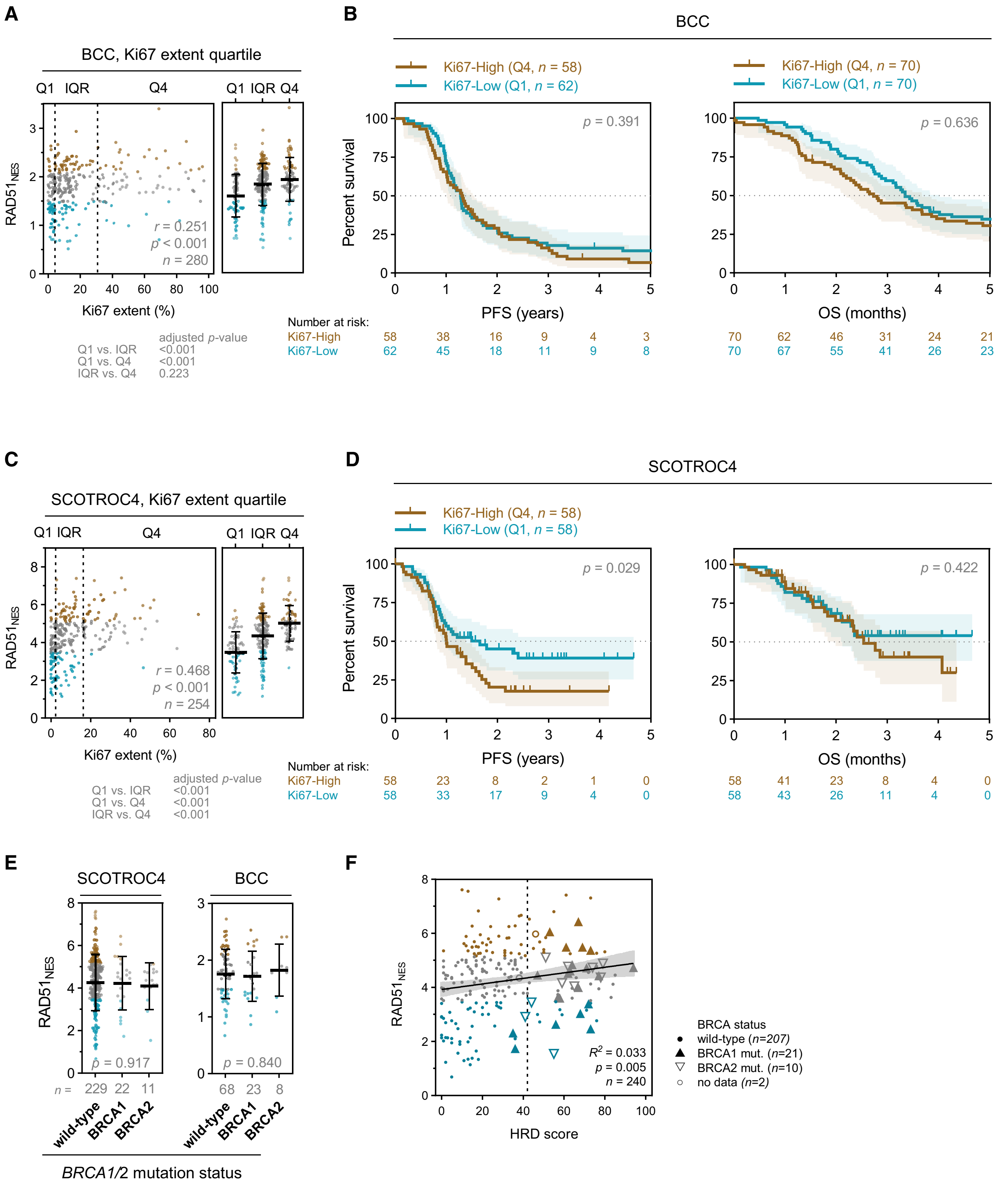

Figure EV2.

 © 2021 The Authors

Figure EV3. Exogenous Flag-RAD51 is functional.

A Immunoblot of RAD51 upon overexpression and subsequent RNAi-mediated depletion of total (siRAD51-CDS) or endogenous (siRAD51-3'UTR) RAD51 mRNA. The exogenous stably overexpressed Flag-RAD51 represents the top band. HGSOC cell utilized indicated below the blot. Blots are representative of two independent experiments.

B Validation of exogenous Flag-RAD51 functionality using a cell viability assay. Flag-RAD51 was stably overexpressed in three HGSOC cell lines which were treated with increasing doses of carboplatin for 96 h. Flag-RAD51 rescues carboplatin sensitivity upon depletion of endogenous RAD51 protein. Mean with standard deviation is shown of at least three biological replicates per point. Statistical comparison is performed at 1μM concentration of carboplatin, *t*-test.

C Immunofluorescence of TYK-nu cells with stable Flag-RAD51 overexpression treated with 10μM of carboplatin for 48 h. Cells were co-stained for both RAD51 and Flag. DAPI serves as a nuclear counterstaining. Scale bar is 20 μm.

© 2021 The Authors

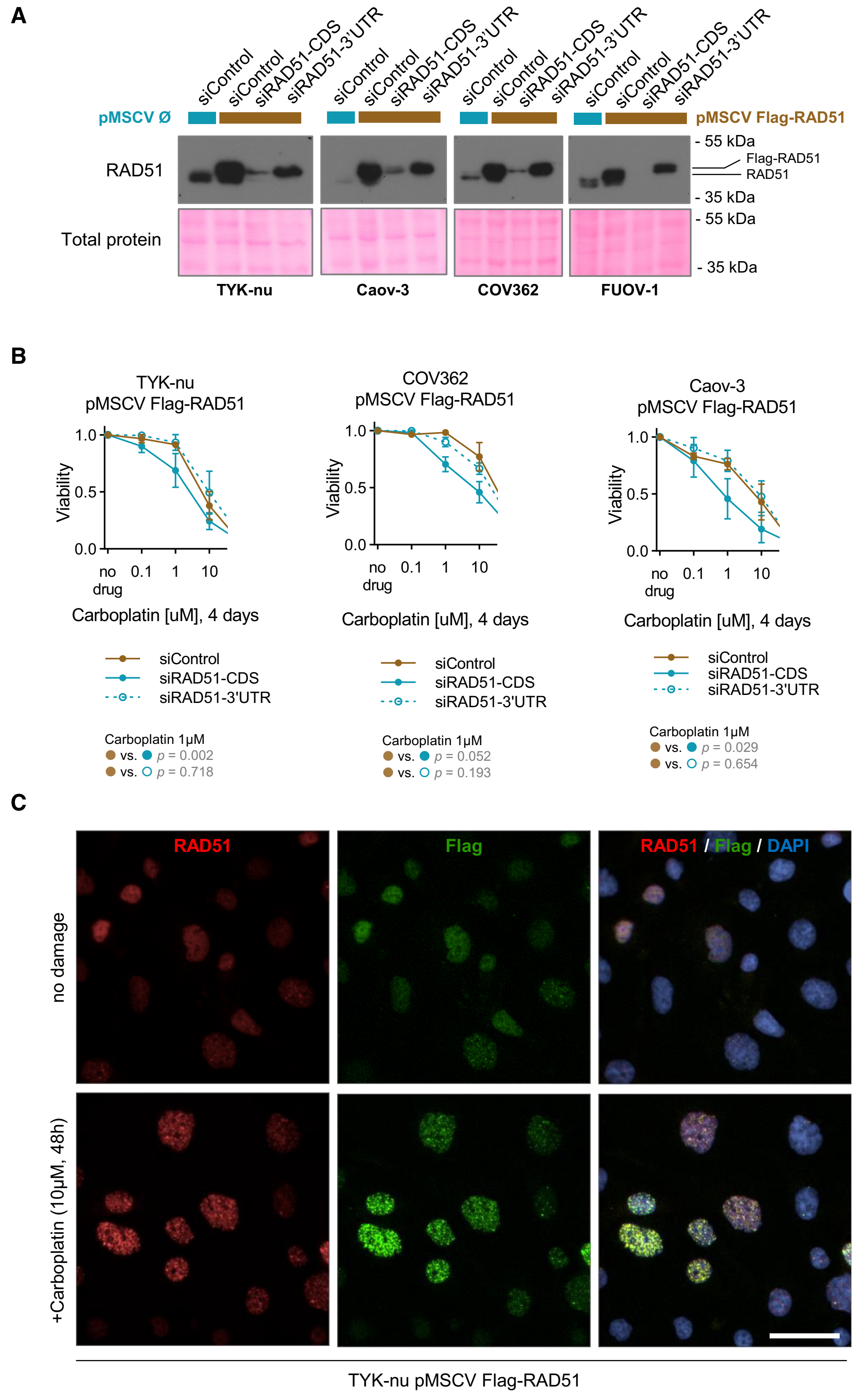

Figure EV3.

© 2021 The Authors

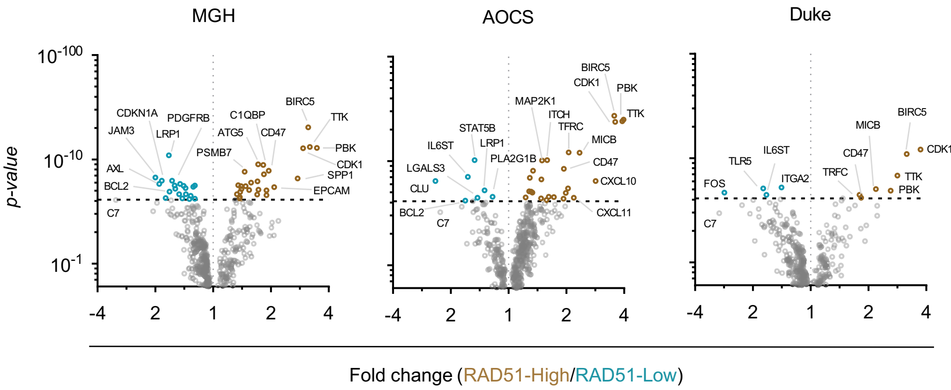

Figure EV4. Differential gene expression analysis of immune genes between RAD51-High and -Low tumours.

Differential gene expression analysis of immune genes between RAD51-High (Q4) and -Low (Q1) across three independent mRNA cohorts of EOC (see also Fig 3E). *t*-Test; dashed line denoted threshold of significance, Bonferroni corrected for multiple testing.

Figure EV5. Multiplexed fluorescent IHC staining for immune markers of the tumour microenvironment.

A Multiplexed fluorescent IHC staining for immune markers of the microenvironment in an EOC patient sample. Unmixed monochrome components are shown along with a false-coloured merge image. Cytokeratin staining was used to differentiate between the tumour and stromal compartments of the sample. Scale bar is 50 μm.
B Quantitation of immune populations in the BCC cohort. Results for RAD51-High and -Low tumours are shown. T/S—tumour/stroma ratio. Bar is median. Mann–Whitney test.
C Subset analysis of $CD8^+$ cytotoxic T-cell infiltration in the BCC cohort stratified according to *BRCA* mutation status. Absolute tumour $CD8^+$ cytotoxic T-cell infiltration numbers and tumour/stroma (T/S) cytotoxic T-cell number ratio in RAD51-High and -Low cases. Bar is median. Mann–Whitney test.

▶

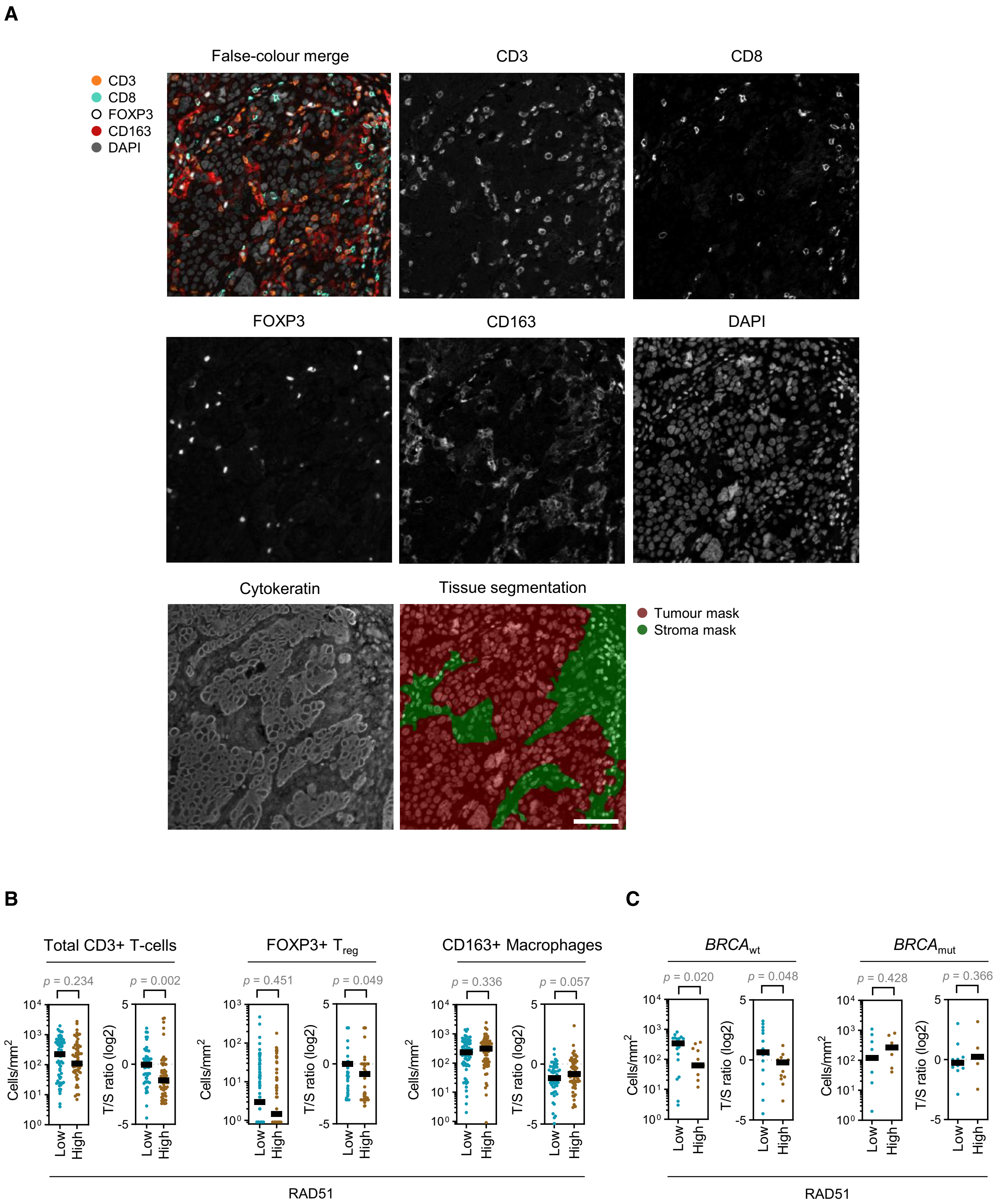

Figure EV5.

 © 2021 The Authors
