# Supplementary material for: Quantitative imaging of RAD51 expression as a marker of platinum resistance in ovarian cancer
Source: EMBO Mol Med. 2021 Mar 11;13(5):e13366. doi: 10.15252/emmm.202013366 (PMC8103098; doi:10.15252/emmm.202013366)
Supplement: Supplementary file 3 — Table EV1 [file EMMM-13-e13366-s006.zip › EMM-2020-13366-V2_TableEV1/EMM-2020-13366-V2_TableEV1README.docx]

Table EV1: GSEA analysis of HGSOC cell lines- RAD51 overexpression vs control
