# Supplementary material for: Quantitative imaging of RAD51 expression as a marker of platinum resistance in ovarian cancer
Source: EMBO Mol Med. 2021 Mar 11;13(5):e13366. doi: 10.15252/emmm.202013366 (PMC8103098; doi:10.15252/emmm.202013366)
Supplement: Supplementary file 6 — Table EV4 [file EMMM-13-e13366-s004.zip › EMM-2020-13366-V2_TableEV4/EMM-2020-13366-V2_TableEV4README.docx]

Table EV4: Differential immune gene expression (RAD51-High Q4 vs RAD51-Low Q1) for four EOC mRNA cohorts: TCGA, AOCS, MGH and Duke
